# Haploinsufficiency of A20 caused by a novel nonsense variant or entire deletion of *TNFAIP3* is clinically distinct from Behçet’s disease

**DOI:** 10.1186/s13075-019-1928-5

**Published:** 2019-06-04

**Authors:** Naomi Tsuchida, Yohei Kirino, Yutaro Soejima, Masafumi Onodera, Katsuhiro Arai, Eiichiro Tamura, Takashi Ishikawa, Toshinao Kawai, Toru Uchiyama, Shigeru Nomura, Daisuke Kobayashi, Masataka Taguri, Satomi Mitsuhashi, Takeshi Mizuguchi, Atsushi Takata, Noriko Miyake, Hideaki Nakajima, Satoko Miyatake, Naomichi Matsumoto

**Affiliations:** 1 Department of Human Genetics, Yokohama City University Graduate School of Medicine, 3-9 Fukuura, Kanazawa-ku, Yokohama, 236-0004 Japan; 2 Department of Stem Cell and Immune Regulation, Yokohama City University Graduate School of Medicine, 3-9 Fukuura, Kanazawa-ku, Yokohama, 236-0004 Japan; 3 Division of Immunology, National Center for Child Health and Development, Tokyo, Japan; 4 Division of Gastroenterology, National Center for Child Health and Development, Tokyo, Japan; 5 Department of Pediatrics, University of Tokyo Hospital, Tokyo, Japan; 6 Division of Clinical Nephrology and Rheumatology, Kidney Research Center, Niigata University Graduate School of Medical and Dental Sciences, Niigata, Japan; 7 Department of Data Science, Yokohama City University School of Data Science, Yokohama, Japan; 8 Clinical Genetics Department, Yokohama City University Hospital, Yokohama, Japan

**Keywords:** *TNFAIP3*, Haploinsufficiency of A20, Behçet’s disease, Whole exome sequencing, Autoinflammatory

## Abstract

**Background:**

Haploinsufficiency of A20 (HA20) is caused by loss-of-function *TNFAIP3* variants. Phenotypic and genetic features of HA20 remain uncertain; therefore, the clinical distinction between HA20 and Behçet’s disease (BD) requires clarification.

**Methods:**

We have collected 12 Japanese BD-like families. Probands of these families were analyzed by whole exome sequencing (WES) and subsequent Sanger sequencing. Clinical features were compared between 54 HA20 patients (including previously reported and new cases) and 520 Japanese BD patients.

**Results:**

We identified c.1434C>A:p.(Cys478*) in one family and a 236 kb deletion at 6q23.3 containing *TNFAIP3* in another family. Four HA20 patients in the two families presented with childhood-onset recurrent oral and genital ulcers and were initially diagnosed and treated as BD. Consistent with the clinical features of HA20, recurrent, refractory fever attacks (three of four patients), and digestive ulcers (two of the four patients) were observed. A comparison of clinical features between HA20 patients and cohorts of BD patients revealed several critical features specific to HA20. These were early-onset, familial occurrence, recurrent fever attacks, gastrointestinal involvement, and infrequent ocular involvement.

**Conclusions:**

We identified a novel nonsense variant and deletion of the entire *TNFAIP3* gene in two unrelated Japanese HA20 families. Genetic screening of *TNFAIP3* should be considered for familial BD-like patients with early-onset recurrent fevers.

**Electronic supplementary material:**

The online version of this article (10.1186/s13075-019-1928-5) contains supplementary material, which is available to authorized users.

## Introduction

Behçet’s disease (BD) is an inflammatory disease of unknown etiology, with recurrent oral and genital ulcers, uveitis, skin inflammation, enterocolitis, and inflammation in other organs [1, 2]. Genome-wide association studies show that common genetic factors are involved in the pathogenesis of the disease [3]. In addition, rare monogenic conditions manifest BD-like phenotypes [4, 5]. Heterozygous loss-of-function *TNFAIP3* variants identified in BD-like patients are now classified as haploinsufficiency of A20 (HA20) [5]. Unlike typical BD, HA20 presents various autoinflammatory and/or autoimmune symptoms in addition to a BD-like phenotype, indicating that there may be HA20-specific symptoms compared with those of BD [5–15]. It is important to accumulate HA20 patients to understand its full clinical spectrum. We here report a novel heterozygous *TNFAIP3* variant and a copy number variation found in two unrelated families. Clinical features of HA20 and BD are discussed.

## Materials and methods

### Patients

A series of families, each with more than two or more patients with BD-like symptoms, were recruited. All patients met the diagnostic criteria (revised in 1987) of the Behçet’s Disease Research Committee, Ministry of Health, Labor and Welfare of Japan [16]. The study protocol was approved by the institutional review boards of Yokohama City University School of Medicine and the National Center for Child Health and Development, and written informed consent was obtained from all patients or their parents. For comparison of clinical features between HA20 and BD, we used a previously described BD cohort from the Yokohama City University Hospital [17].

### Whole-exome sequencing

Peripheral-blood leukocytes from affected individuals and their families were collected. Genomic DNA was extracted using QuickGene-610 L (Fujifilm, Tokyo, Japan) according to the manufacturer’s protocol. Genomic DNA was sheared and captured using a SureSelect Human All Exon V6 Kit (Agilent Technologies, Santa Clara, CA, USA) and sequenced on a HiSeq2500 or Novaseq 6000 system (Illumina, San Diego, CA, USA) with 101-bp paired-end reads. Exome data processing, variant calling, and annotation were performed as previously described [18]. In brief, reads were aligned to GRCh37 with Novoalign (http://www.novocraft.com/), and PCR duplicates were removed using Picard (http://broadinstitute.github.io/picard/). Local realignments around indels and base quality-score recalibration were performed using the Genome Analysis Toolkit (GATK). Variants were called by the GATK UnifiedGenotyper and filtered according to GATK Best Practices (version 3) (https://software.broadinstitute.org/gatk/). The common variants registered in dbSNP137 (minor allele frequency ≥ 0.01) without known clinical associations were excluded from further analysis. Included variants were annotated using ANNOVAR (http://annovar.openbioinformatics.org/). The mean depth of coverage against the RefSeq coding sequence (CDS) was 64.7×, and 97.0% of CDS was covered by 10 reads or more. To identify causal variants, the obtained variants were filtered according to the following exclusion criteria: (a) variants with a > 1% minor allele frequency in the Exome Aggregation Consortium database (ExAC, Cambridge, MA, http://exac.broadinstitute.org/), (b) variants observed in 575 Japanese in-house control exomes, and (c) synonymous variants. We evaluated the remaining variants under the assumption of autosomal dominant inheritance and particularly focused on rare variants in genes known to be involved in autoinflammatory diseases. Variants and their familial segregation were confirmed using Sanger sequencing.

Copy number variants (CNVs) were examined using whole-exome sequencing (WES) data as previously described [19, 20]. Two algorithms were used: the eXome-Hidden Markov Model (XHMM) [21] and a program based on the relative depth of coverage ratios developed by Nord et al. [22], hereafter called Nord’s method. In brief, XHMM detects CNVs from entire coding regions by analyzing normalized raw exome read depth data with principal component analysis (PCA) and the hidden Markov model. Nord’s method evaluates targeted genes using raw exome read depth data. Candidate CNVs were validated by quantitative PCR.

### Reverse transcription polymerase chain reaction

Lymphoblastoid cell lines derived from patient 1 and 2 were grown in Roswell Park Memorial Institute 1640 medium supplemented with 10% fetal bovine serum, tylosin and antibiotic-antimycotic solution at 37 °C in a 5% CO_2_ incubator. After incubation with dimethyl sulfoxide (DMSO) (as vehicle control) or 30 μM cycloheximide (CHX) to observe the preventive effects of CHX on nonsense-mediated mRNA decay (NMD) for 4 h, total RNA was extracted using an RNeasy Plus Mini Kit (QIAGEN, Hilden, Germany). cDNA was synthesized from 2.5 μg of total RNA using random hexamers and the SuperScript III First-Strand Synthesis System (Invitrogen, Carlsbad, California). The control cDNA was isolated from a patient with a different disease (early-onset epilepsy). PCR included 24 cycles with specific primers for the exon 6–7 boundary and the exon 7–8 boundary (available on request). PCR products electrophoresed in 2% agarose gel were stained with ethidium bromide. PCR was conducted with the autosomal internal control locus (*ACTB*). RT-PCR products were sequenced by the Sanger method.

### Statistical analysis

Statistical analysis was performed with SPSS version 22 (IBM Japan, Tokyo, Japan). Categorical variables were analyzed using the chi-square test. Continuous variables were examined using Student’s *t* test. A *p* value less than 0.05 was considered statistically significant.

## Results

### Overview of the studied families

Twenty-five patients from 12 families were collected (data not shown). In each family, WES was performed on the proband and on other selected family members. Two novel *TNFAIP3* pathogenic changes were found in two families (16.7%, 2 of 12).

### Identification of *TNFAIP3* variants

**Fig. 1:**
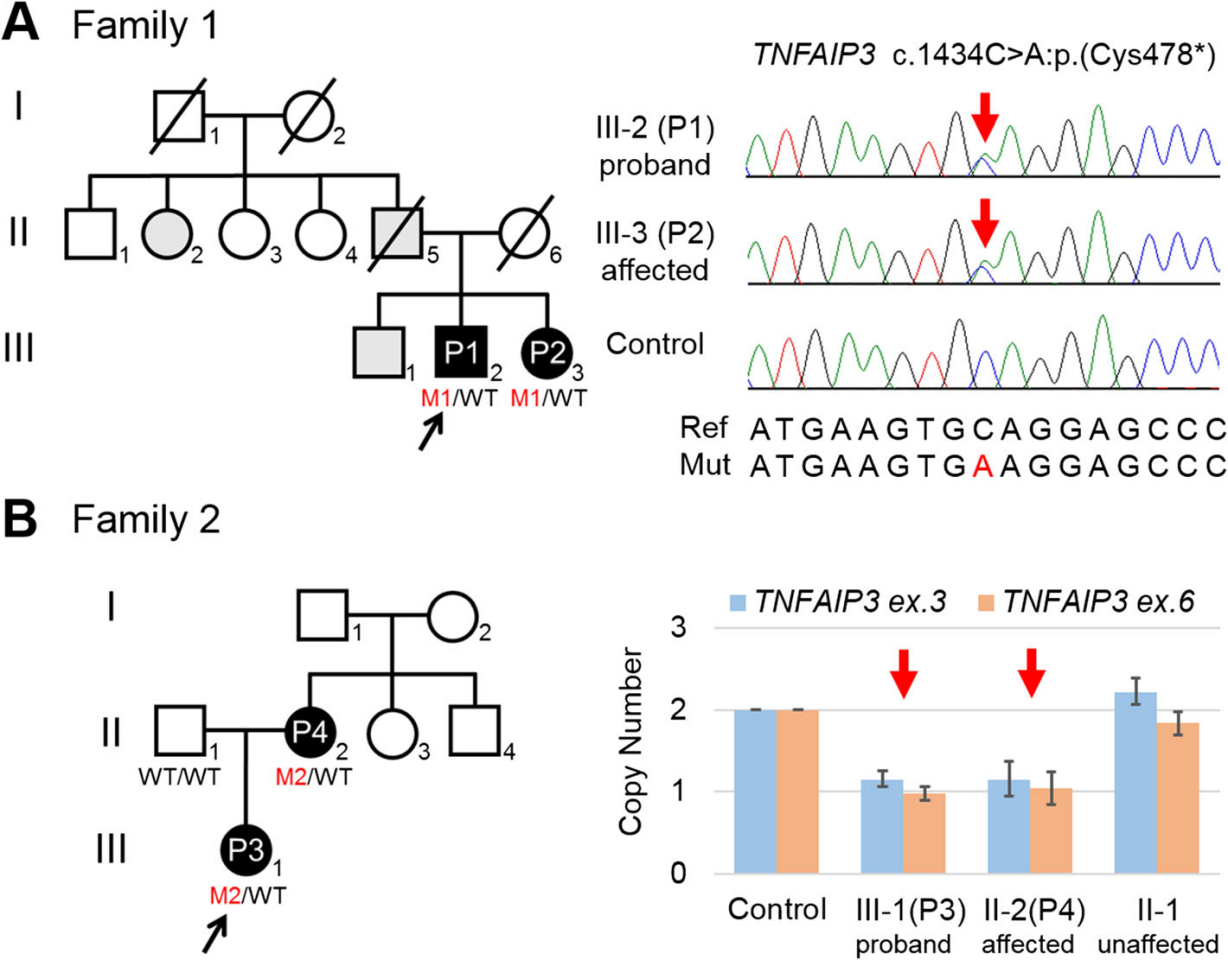
*TNFAIP3* variants in two HA20 families. **a** Pedigree of family 1 and electropherograms of two patients with a *TNFAIP3* variant and a control subject. **b** Pedigree of family 2 and quantitative PCR of family members with a *TNFAIP3* deletion. Affected individuals and probably affected individuals are depicted using black and gray symbols, respectively. Black and red arrows indicate the probands and variants, respectively. The *TNFAIP3* alleles identified in each individual are labeled as M1 and M2 for variants and WT for wild-type. M1: c.1434C>A:p.(Cys478*), M2: chr6:138192201-138428412 (GRCh37/hg19) deletion

WES was performed on the probands (patient 1 from family 1 and patient 3 from family 2) and on the parents of patient 3 (Fig. 1a, b).

**Fig. 2:**
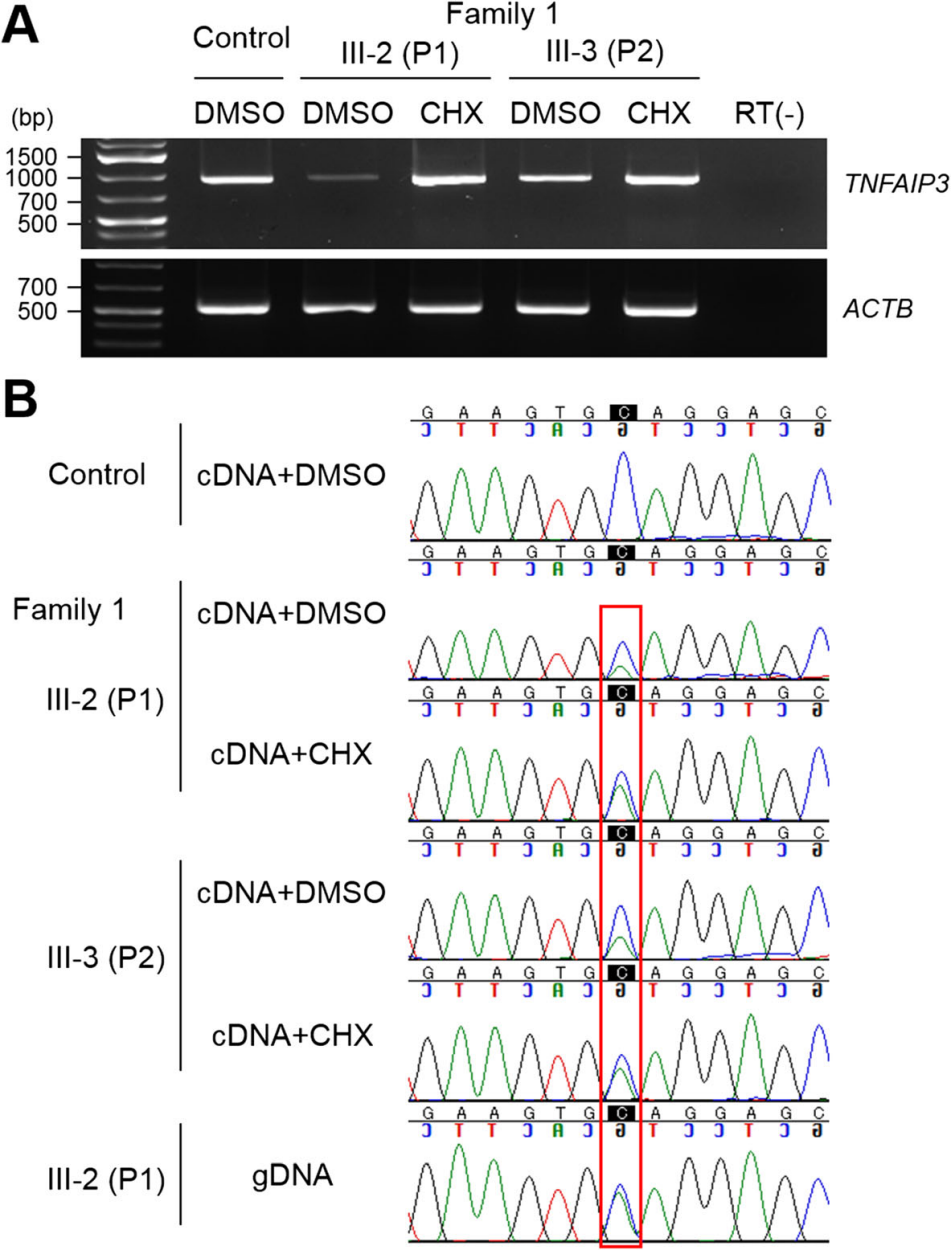
RT-PCR analysis of lymphoblastoid cell lines from patients 1 and 2 in family 1. Total RNA was extracted from a patient-derived lymphoblastoid cell line treated with or without CHX. **a** Electrophoresis of RT-PCR products. The band intensity in cells from affected patients treated with DMSO (vehicle control) was weak compared to that of control cells and was significantly stronger after CHX treatment (an NMD inhibitor). **b** Electropherograms of RT-PCR products (reverse strand). The mutant allele was recovered by CHX treatment. Red square highlights the change in signal of the mutant allele. CXH cycloheximide, DMSO dimethyl sulfoxide, NMD nonsense-mediated mRNA decay, RT (−) without reverse transcriptase (negative control)

In patient 1, we identified a novel nonsense variant, c.1434C>A:p.(Cys478*), in *TNFAIP3* (NM_006290.3). This variant was absent from ExAC, in-house exome controls, and Infevers (an online database for autoinflammatory mutations, https://infevers.umai-montpellier.fr/web). We detected no variants in other genes associated with autoinflammatory diseases. Sanger sequencing confirmed the same variant in the affected sister (patient 2) (Fig. 1a). To observe mutational effects of this variant, RT-PCR was performed using total RNA extracted from lymphoblastoid cell lines derived from patients 1 and 2. RT-PCR products were electrophoresed and the band intensity in DMSO-treated cells from affected patients was weak compared to that from DMSO-treated control cells. Band intensity was stronger after CHX treatment (Fig. 2a). Direct sequencing of PCR products showed that CHX treatment increased the presence of the mutant allele compared with DMSO treatment (Fig. 2b). These results indicate that this nonsense variant may be subjected to NMD [23]. Based on the American College of Medical Genetics and Genomics guidelines [24], this variant is classified as pathogenic (PVS1, PM2, PP3, PP4).

**Fig. 3:**
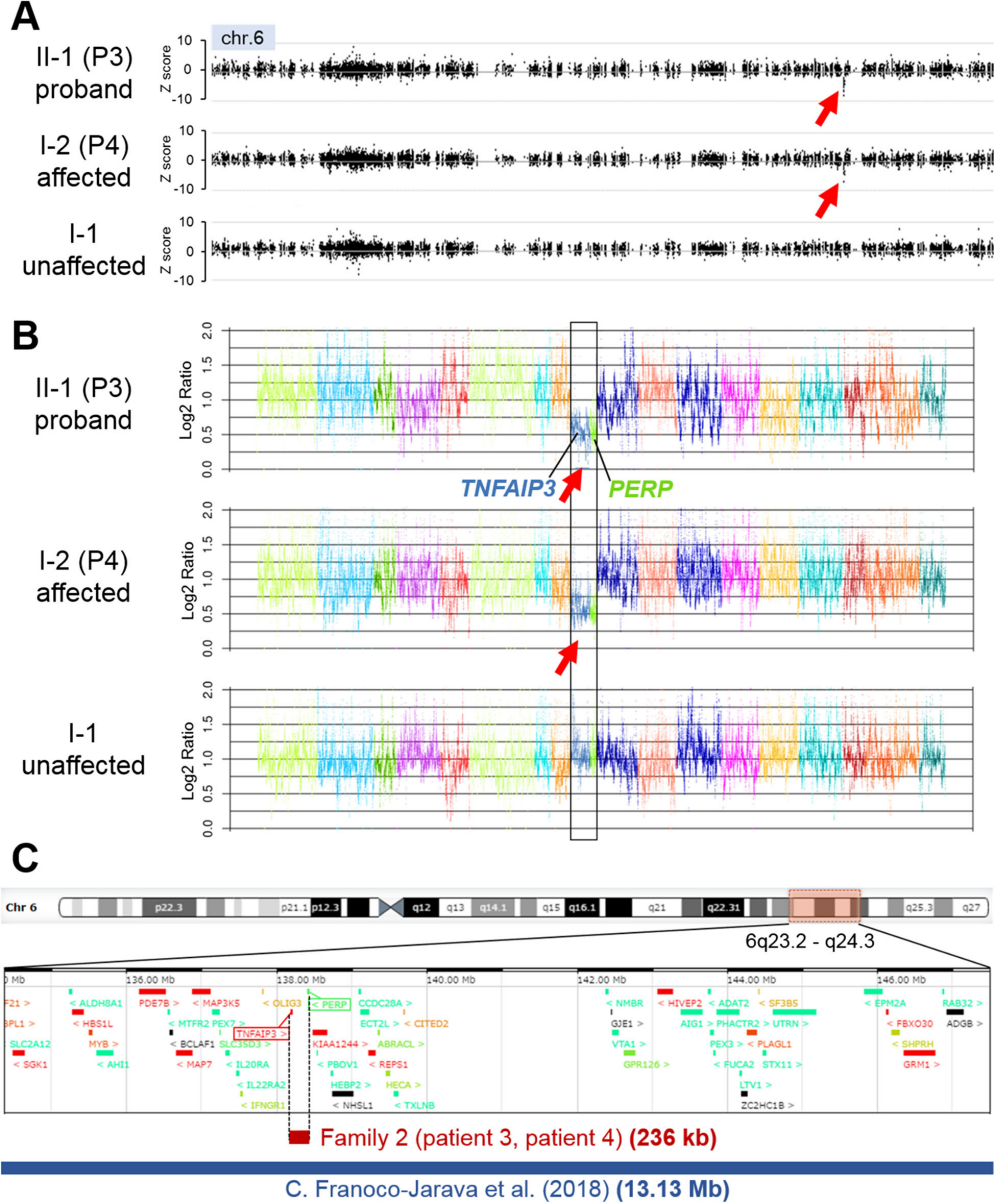
CNV analysis by XHMM and Nord’s method in family 2. **a** A 236 kb deletion at 6q23.3 involving *TNFAIP3* in patients 3 and 4 detected by XHMM. The *x*-axis shows the genomic position, and the *y*-axis indicates the *Z* score. Red arrows represent calls for copy number losses. **b** A deletion involving *TNFAIP3* and *PERP* in patients 3 and 4 detected by Nord’s method. The *x*-axis shows arrays of targeted genes of different colors with their proportional physical length and the *y*-axis shows log_2_ ratios for each targeted base in the genes tested. Red arrows represent calls for copy number losses. **c** Schematic representation and comparison of the deletions containing *TNFAIP3*. The thick red bar represents the 236 kb deletion in family 2, and the thick blue bar represents the 13 Mb deletion at 6q23.2-q24.3 reported by Franoco-Jarava et al. (2018). The genomic region diagrams were captured from the browser using DECIPHER (http://decipher.sanger.ac.uk). CNV copy number variation, XHMM eXome Hidden Markov Model

In family 2, no candidate single nucleotide variant or small indel was found in any gene associated with autoinflammatory diseases. WES-based CNV analysis identified a 236 kb deletion at 6q23.3 [chr6:138192201-138428412 (GRCh37/hg19)] involving *TNFAIP3* and *PERP* in patients 3 and 4 (Figs. 1b and 3a, b). Several CNVs involving *TNFAIP3* were reported in the Database of Genomic Variants (DGV) (http://dgv.tcag.ca/dgv/app/), ExAC, and DECIPHER (http://decipher.sanger.ac.uk), but these CNVs are not common in the Japanese population. Five patients in DECIPHER have a > 4 Mb deletion involving *TNFAIP3* and more than 30 genes. Intellectual disability was recorded for two of these patients, but no information regarding inflammatory status was recorded. In addition, four healthy individuals registered in DGV and ExAC have deletions involving *TNFAIP3*; three cases have a *TNFAIP3* deletion only and the other case has a 934 kb deletion involving *TNFAIP3* and four other genes. Familial HA20 clearly indicates reproductive fitness [5–7, 11–14], and the penetrance of *TNFAIP3* abnormality could be variable. Recently, a HA20 patient was described with a 13-Mb CNV encompassing *TNFAIP3* and another 52 genes (Fig. 3c) [8]. Meanwhile, germline *PERP* pathogenic variants have never been reported in any human diseases. In the animal model, *Perp* knockout mice showed postnatal lethality and defects in the skin and other ectodermal derivatives, resembling ectodermal dysplasia syndrome [25]. *Perp* heterozygous mice showed no expected clinical phenotypes [26]. Patients in family 2 showed no signs of ectodermal dysplasia. There are small numbers of truncating variants in a healthy public database, ExAC, and DGV. Quantitative PCR confirmed the deletion in the affected proband and her mother (Fig. 1b). Considering these findings, the phenotype of the affected patients in family 2 can be explained by *TNFAIP3* haploinsufficiency.

### Clinical features

The clinical features of the two families with *TNFAIP3* variants are summarized in Table 1.

**Table 1:** Summary of patients with *TNFAIP3* variants

Family	Family 1		Family 2	
Patient	Patient 1	Patient 2	Patient 3	Patient 4
Age	35 years	34 years	12 years	42 years
Gender	M	F	F	F
Detected variant	*TNFAIP3* (NM_006290.3) c.1434C>A:p.(Cys478*)		chr6:138192201-138428412 (GRCh37/hg19) deletion (including *TNFAIP3*)	
Initial symptoms	Fever, lymphadenopathy	Oral ulcer, genital ulcer	Periodic fever	Oral ulcer
Symptom onset	6 years	12 years	2 months	1 year
Age at diagnosis	7 years	19 years	11 years	16 years
Fever	+	+	+	–
Lymphadenopathy	+	+	+	+
Oral ulcers	+	+	+	+
Genital ulcers	+	+	+	+
Skin lesions	–	Erythema nodosum, pernio-like rash	–	Folliculitis
Ophthalmic signs	–	–	Acute anterior uveitis	–
Digestive signs	Nausea	Abdominal pain, vomits, digestive ulcers, colitis	Abdominal pain, diarrhea, bloody stools, weight loss, digestive ulcers	Abdominal pain
Musculoskeletal signs	–	–	+	–
Others	Pharyngalgia	Raynaud’s phenomenon	Pharyngalgia, enlarged tonsil	Thyroiditis
Treatment	PSL, colchicine	Symptomatic treatment, PSL, colchicine	Colchicine, cimetidine, PSL, mesalazine, NSAIDs, MTX, corticosteroid eye drops	Levothyroxine

*NSAIDs* non-steroidal anti-inflammatory drugs, *MTX* methotrexate, *PSL* prednisolone

#### Family 1

Patient 1 (III-2 in Fig. 1a) is a 35-year-old male who is the second child of non-consanguineous Japanese parents. He presented with fever and lymphadenopathy at the age of 6. He was diagnosed with BD at 7 years of age because of recurrent oral and perianal ulcers and was prescribed with oral prednisolone (PSL). He had recurrent episodes of high-grade fever (up to 39 °C) associated with lymphadenopathy, pharyngalgia, and nausea. PSL dosage was adjusted according to the patient’s condition, and the withdrawal of PSL was difficult. He did not show any ophthalmological or neurological symptoms. He is currently treated with PSL (12.5 mg/day) and colchicine (1.0 mg/day).

**Fig. 4:**
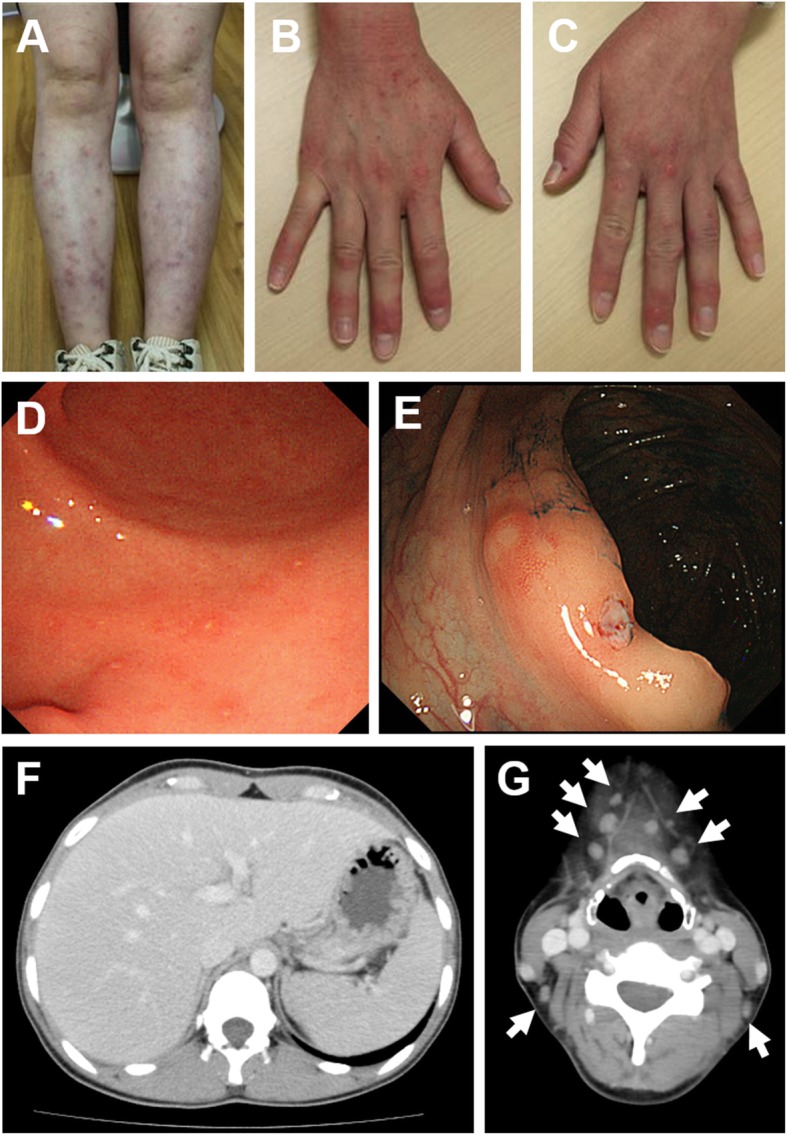
Skin and gastrointestinal lesions of patient 2. **a** Erythema nodosum in the bilateral lower extremities. **b**, **c** Pernio-like rash in the fingers. **d**, **e** Endoscopic findings at 29 years of age; **d** multiple ulcers in the gastric mucosa and **e** ulceration in the ileocecum. **f**, **g** CT findings at 29 and 33 years of age; **f** hepatosplenomegaly and **g** generalized lymphadenopathy. White arrows indicate swollen lymph nodes

Patient 2 (III-3 in Fig. 1a) is the proband’s younger sister. She had oral and genital ulcers at 12 and 15 years of age, respectively. At the age of 19, she presented with fever, oral, and genital ulcers, and she was diagnosed with BD. At 25 years of age, she was hospitalized due to fever and erythema nodosum and she was treated with PSL and colchicine. Thereafter, she was treated with low-dose PSL (5–10 mg/day) and colchicine, but they were ineffective in preventing further attacks. At 29 years of age, she presented with Raynaud’s phenomenon. Skin biopsy of erythema nodosum (Fig. 4a) showed similar pathological findings to cutaneous periarteritis nodosa or thrombophlebitis. Gastrointestinal endoscopy revealed multiple ulcers in the stomach and colon, which were not typical of intestinal BD (Fig. 4d, e). Torso CT imaging showed hepatosplenomegaly and generalized lymphadenopathy (Fig. 4f). She had bouts of recurrent fever, erythema nodosum, cervical lymphadenopathy, and abdominal pain lasting 2 weeks several times a year, but these symptoms subsided spontaneously without any treatment. At 33 years of age, a CT scan confirmed persisting hepatosplenomegaly and lymphadenopathy (Fig. 4g) and she presented with a severe pernio-like rash in the cold season (Fig. 4b, c). Aspiration biopsy of cervical lymph nodes showed no signs of malignant lymphoma.

The proband’s elder brother (III-1 in Fig. 1a) had multiple episodes of hospitalization due to the fever of unknown origin, but detailed clinical information is not available. The proband’s father (II-5 in Fig. 1a) had recurrent oral ulcers. At 58 years of age, he presented with intractable skin lesions and poly-lymphadenopathy. Lymph node biopsy showed reactive inflammation, but no immuno-suppressants were needed for treatment. At 59 years of age, he was diagnosed with amyotrophic lateral sclerosis and passed away from aspiration pneumonia at the age of 60. The proband’s mother (II-6 in Fig. 1a) had no BD-like symptoms and passed away from colon cancer. The proband’s aunt on the paternal side (II-2 in Fig. 1a) had recurrent febrile episodes, but details are unavailable.

#### Family 2

**Fig. 5:**
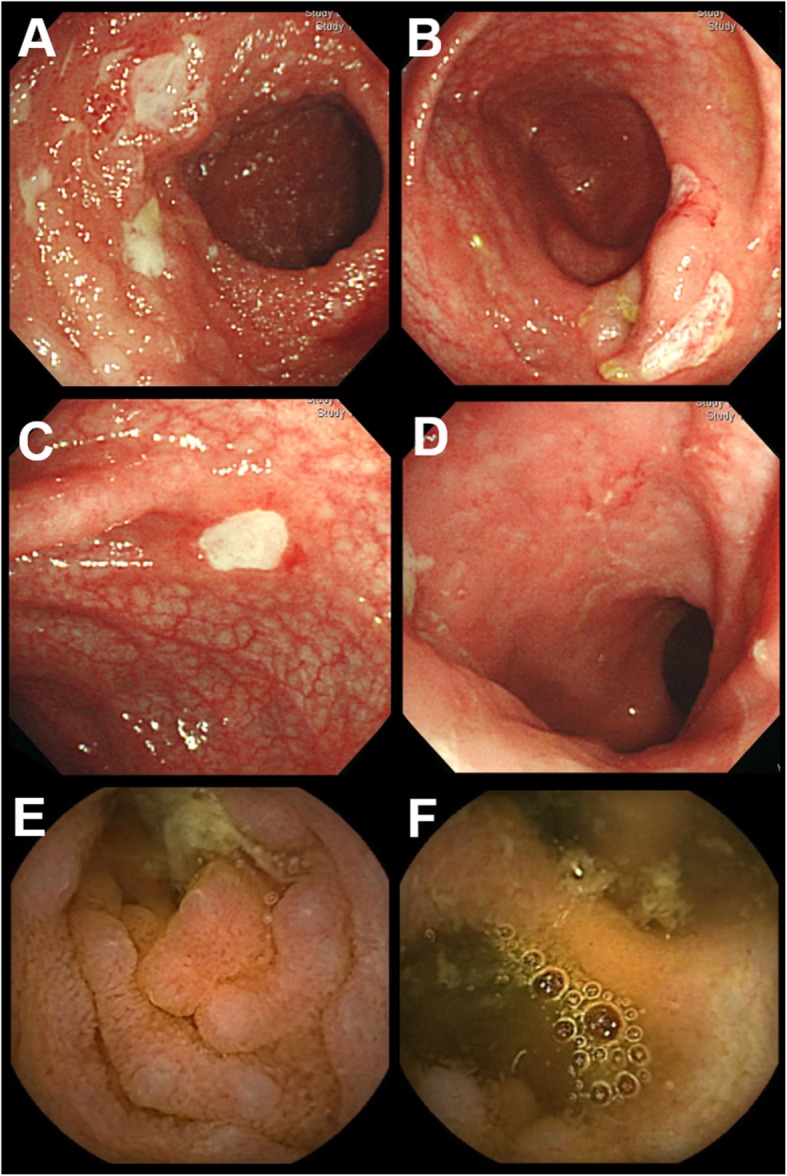
Endoscopic findings of patient 3 at 10 years of age. Multiple ulcers were detected throughout the intestinal tract by endoscopy; **a** terminal ileum, **b** cecum, **c** transverse colon, **d** rectum, **e** duodenum, and **f** ileum. Most of the mucosa surrounding ulcerous lesions was normal, but there was mild inflammation in the rectal mucosa

Patient 3 (III-1 in Fig. 1b) is a 12-year-old girl who is the first child of non-consanguineous Japanese parents. At 2 months of age, she presented with 38–39 °C fever accompanied by abdominal pain, diarrhea, ankle arthralgia, oral ulcers, pharyngalgia, and an enlarged tonsil. Febrile attacks were recurrent every 1–2 weeks and resolved within 4 days. She presented with perianal ulcers (at 4 years old), bloody stool, and weight loss (at 5 years old), but colonoscopy did not identify any abnormality. At 9 years of age, she was prescribed with naproxen and methotrexate (MTX) because of left ankle arthritis, but MTX was stopped because of abdominal pain. She was treated with colchicine and mesalazine for abdominal pain, but they were not effective. At 10 years of age, she was suspected of having a periodic fever, aphthous stomatitis, pharyngitis, and adenitis (PFAPA) syndrome and was treated with cimetidine, which contributed to a reduction in the number of febrile attacks. Gastrointestinal and capsule endoscopy revealed multiple ulcers throughout the intestinal tract (Fig. 5), and mesalazine was re-administered. Histopathology of ulcer biopsies showed infiltration of lymphocytes and plasma cells. There was no evidence of granuloma, cryptitis, or crypt abscess, and the findings were thought to indicate nonspecific chronic inflammation. At 11 years of age, bilateral non-granulomatous acute anterior uveitis was revealed by ophthalmologic examination and diagnosed as BD. She did not show any neurodevelopmental delay. Currently, she experiences genital ulcers and has been treated with colchicine (400 mg/day), cimetidine (1.0 mg/day), and corticosteroid eye drops. The severity of abdominal pain is milder than before and fever is less frequent.

Patient 4 is the proband’s mother (II-2 in Fig. 1b). At 1 year of age, she presented with intractable oral ulcers and was twice admitted to hospital for dehydration. At 12 years of age, she had oral ulcers during orthodontic treatment. At 15 years of age, she presented with genital ulcers and abdominal pain. At 16 years of age, she was diagnosed with BD. Colonoscopy at 18 years of age did not show any abnormality. From 19 years of age, her symptoms were in remission and she did not require treatment. At 34 years of age, she showed folliculitis at the lower limbs. At 35 years of age, she was diagnosed with Hashimoto disease and was treated with levothyroxine. She presented fever with tonsillitis and lymphadenitis several times a year since her childhood, but they were not apparently periodic nor present in prolonged courses. She did not show any ophthalmological, neurological, and musculoskeletal symptoms.

The other maternal relatives (I-1, I-2, II-3, and II-4 in Fig. 1b) had no BD-like symptoms.

#### Comparison of HA20 and BD clinical features

Our study raises the possibility that some BD patients may actually be HA20 patients because our two HA20 families had long been diagnosed and treated as BD. If BD and HA20 treatment strategies are different, the precise diagnosis of HA20 and differentiation from typical BD is important. We utilized the data from a large cohort of BD patients accumulated at our institute [17] to conduct a comparison of the clinical features of HA20 and BD.

Table 2 summarizes the clinical features of 54 cases with *TNFAIP3* variants (50 previously reported cases [5–15] and the four cases described here) and compares these with those of our 520 archived BD cases. Consistent with previous reports [6–8], recurrent oral and genital ulcers, skin legions, gastrointestinal, and musculoskeletal involvement were frequent in HA20. These clinical symptoms are similar to those of BD, and it is reasonable that 51.9% of HA20 cases were initially diagnosed as BD. However, HA20 showed significantly earlier onset (6.0 ± 6.5 years) and higher familial occurrence (60.0% in pedigrees). Clinically, HA20 showed significantly more recurrent fever (72.5%), gastrointestinal involvement (41.5%), and less eye involvement (9.6%) compared with BD. Among HA20 patients with gastrointestinal involvement, 18 of 22 patients (81.8%) showed gastrointestinal ulcers, including two cases with BD-typical ulcers, and ulcers were distributed in various regions of the digestive tract. Detailed information and images of gastrointestinal lesions were not available for all previous cases, and thus, it was difficult to investigate additional features. Some patients did not undergo a gastrointestinal scan, but 34 of the 53 HA20 patients presented with some gastrointestinal symptoms. The positive rate of HLA-B51 in HA20 patients was lower (27.3%) than that in BD (47.8%), but because the majority of HA20 has been reported in European populations whose HLA-B51 positive rate is lower than that of Japanese populations, it is uncertain whether HLA-B51 affects the HA20 phenotype. Concomitant autoimmune disease and positive autoantibodies were suggested in previous reviews [6, 7], and indeed, there was a trend for this compared with our BD cohort, but analysis of additional HA20 cases is needed for confirmation.

**Table 2:** Clinical features of haploinsufficiency of A20 (HA20) and Behçet’s disease (BD)

Characteristics	HA20^5–15^ (*n* = 54^a^)	(%)	BD (*n* = 520)	(%)	*p*	Odds ratio	95% CI	
Age at onset^b^ (years) (mean ± SD)	6.0 ± 6.5		36.4 ± 12.3		*< 0.001* ^f^			
Childhood onset (< 16 years old)	49/53	92.5	10/520	1.9	*< 0.001*	624.75	188.92	2065.98
Observation period (years) (mean ± SD)	15.5 ± 14.9		13.7 ± 12.0		0.37^f^			
Gender male	20/54	37.0	241/520	46.3	0.19	0.68	0.38	1.22
Familial^c^	15/25	60.0	19/332	5.7	*< 0.001*	24.71	9.80	62.29
Recurrent fever	37/51	72.5	39/364	10.7	*< 0.001*	22.02	10.99	44.30
Oral ulcer	46/52	88.5	518/520	99.6	*< 0.001*	0.03	0.01	0.15
Genital ulcer	34/52	65.4	372/520	71.5	0.35	0.75	0.41	1.37
Eye involvement	5/52	9.6	330/520	63.5	*< 0.001*	0.06	0.02	0.16
Skin involvement	28/52	53.8	461/520	88.7	*< 0.001*	0.15	0.08	0.27
Arthritis	21/54	38.9	245/520	47.1	0.25	0.71	0.40	1.27
Gastrointestinal involvement^d^	22/53	41.5	78/520	15.0	*< 0.001*	4.02	2.21	7.31
Vascular involvement	7/53	13.2	41/520	7.9	0.18	1.78	0.76	4.19
CNS involvement	5/53	9.4	57/520	11.0	0.73	0.85	0.33	2.21
Autoimmune diseases^e^	26/54	48.1	36/520	9.1	*< 0.001*	9.26	4.91	17.46
Anti-nuclear antibody (≧160×)	3/12	25.0	15/288	5.2	*0.03*	6.07	1.49	24.76
HLA-B51	3/11	27.3	195/408	47.8	0.23	0.41	0.11	1.57
Past/current colchicine use	29/53	54.7	374/518	72.2	*0.008*	0.47	0.26	0.83
Past/current bDMARDs use	19/53	35.8	89/520	17.1	*0.001*	2.71	1.48	4.96
Fulfilling ISG criteria for BD	23/54	42.6	468/520	90.0	*< 0.001*	0.08	0.05	0.15

*BD* Behçet’s disease, *bDMARDs* biological disease modifying anti-rheumatic drugs, *CNS* central nervous system, *HA20* haploinsufficiency of A20, *ISG* International Study Group. Significant results are highlighted in italics

^a^Includes previously reported^5–15^ and current cases with genetically confirmed HA20

^b^Age at “symptom onset” for HA20 and “diagnosis” for BD

^c^Ratio of the pedigree with familial aggregation among all families

^d^Cases with gastrointestinal lesions revealed by imaging (endoscope, CT)

^e^Autoimmune diseases including systemic diseases (rheumatic diseases) and organ-specific diseases (e.g.,. Hashimoto disease, insulin-dependent diabetes, etc.)

^f^Analyzed using the unpaired *t* test. Other variables were analyzed using the chi-square test

Considering the difference in ethnicity among HA20 and our BD cohort, we conducted sub-analysis of confined Japanese patients with HA20 and BD, which revealed similar results (Additional file 1: Table S1), while it is well known that adult and pediatric BD exhibit distinct clinical features. Because HA20 showed more childhood-onset, we conducted an additional comparative analysis of HA20 and pediatric BD using data from the Pediatric Behçet’s disease (PEDBD) study [27]. Additional file 2: Table S2 summarizes clinical features. Similar to comparison with adult BD cohort, HA20 showed significantly more familial occurrence, fever, gastrointestinal involvement, and less eye involvement. HA20 cases showed less fulfilling ISG and PEDBD criteria for BD. Our conclusion did not alter by the additional analyses.

## Discussion

In the current study, we identified a novel nonsense variant and a novel CNV in *TNFAIP3* in two families who had been diagnosed as BD. We then compared clinical features in HA20 and BD patients and identified several key features differentiating HA20 from BD. Analyzing *TNFAIP3* is important if HA20 is suspected in BD-like patients.

Comparison of the clinical features between HA20 and BD cohorts revealed that some features are shared between HA20 and BD as previously reported [6–8]; recurrent oral and genital ulcers, and skin, eye, musculoskeletal, and gastrointestinal involvement were commonly seen in both HA20 and BD. Moreover, we identified features that are more likely to occur in HA20 than BD: early-onset disease, family history, recurrent fever, frequent gastrointestinal involvement, and less frequent eye involvement. Applying these criteria, the clinical characteristics of our four patients were more consistent with HA20 than BD.

Only a few years have passed since HA20 was first recognized as a distinct disease; therefore, a diagnosis of HA20 is not easy. Many clinical features of HA20 are similar to those of BD, and about half of HA20 cases were initially diagnosed as BD [5–7, 12–14]. However, HA20 is not commonly seen in adult-onset BD (1/768, 0.13%) [5], and our clinical comparison of HA20 and BD indicates further differences (Table 2); therefore, HA20 analysis may only be required in cases with HA20-specific signs, including familial occurrence, early-onset, and/or recurrent fever. This distinction will be important if treatment strategies specific to HA20 will be established. Although anti-cytokine therapy was effective in several cases, there were cases in which only follow-up or low-dose steroids and colchicine were needed. Disease severity and treatment responses vary; therefore, the accumulation of cases with *TNFAIP3* variants is needed to elucidate the pathophysiology and treatment strategies for HA20.

A patient with a 13 Mb deletion involving *TNFAIP3* presenting autoinflammation and psychomotor and growth delay was reported [8]. This 13 Mb CNV contains at least 53 protein-coding genes, six of which are linked to the immune system, suggesting that some of these genes affect the phenotype of patients, in addition to *TNFAIP3*. In contrast, the 236 kb CNV in family 2 included only two genes (Fig. 3c), and the affected proband symptoms were consistent with HA20. Therefore, *TNFAIP3* haploinsufficiency resulted in familial HA20. Next-generation sequencing (NGS) data is useful to detect small CNVs that might be difficult to identify by conventional microarray analysis [19–22]. Patient 3 in family 2 did not show neurodevelopmental delay; therefore, microarray and karyotype analysis was not performed. Such a 236 kb deletion may be easily overlooked if careful CNV investigation is not considered.

Finally, there are some limitations to this study. We have investigated mainly familial BD cases by WES analysis but no sporadic cases. Some sporadic HA20 cases with variable phenotypes have been reported [5, 8–12]. Therefore, we may have underestimated the incidence of HA20 among our sporadic BD cohort. It was, however, reported that targeted resequencing of 384 sporadic Japanese BD patients identified no *TNFAIP3* variants [5]. We also detected only two families with HA20 among our 12 BD-like families. Recently, some autoinflammatory-associated genes other than *TNFAIP3* were identified in familial cases with overlapping phenotypes [4, 28, 29]. There must be other genetic abnormalities in other BD families, and they require further thorough investigation. Although BD has a complex, multifactorial genetic etiology, elucidation of the pathophysiology of monogenic autoinflammatory diseases might help to determine the disease mechanisms of BD. Lastly, as our BD cohort is mostly Japanese (98.5%), it is likely that HA20-specific features, relative to BD, may be different in different populations.

In conclusion, we identified a novel *TNFAIP3* single nucleotide variant and a CNV involving *TNFAIP3* in two Japanese families. In patients with HA20-specific features, *TNFAIP3* analysis should be considered, including CNV analysis.

## Additional files

Additional file 1: **Table S1.** Clinical features of Japanese haploinsufficiency of A20 (HA20) and Japanese Behçet’s disease (BD). (DOCX 22 kb)

Additional file 2: **Table S2.** Clinical features of haploinsufficiency of A20 (HA20) and the Pediatric Behçet’s disease (PEDBD). (DOCX 21 kb)

## Data Availability

The datasets used and analyzed during the current study are available from the corresponding author upon reasonable request.

## Abbreviations

BD: Behçet’s disease
CNV: Copy number variation
ExAC: Exome Aggregation Consortium database
GATK: Genome Analysis Toolkit
HA20: Haploinsufficiency of A20
IBD: Inflammatory bowel disease
MTX: Methotrexate
NGS: Next-generation sequencing
NMD: Nonsense-mediated mRNA decay
PFAPA: Periodic fever, aphthous stomatitis, pharyngitis, and adenitis
WES: Whole-exome sequencing
XHMM: eXome-Hidden Markov Model

## References

[1] Sakane T, Takeno M, Suzuki N, Inaba G (1999). Behcet’s disease. N Engl J Med.

[2] International Study Group for Behcet's Disease (1990). Criteria for diagnosis of Behcet’s disease. Lancet.

[3] Kirino Y, Bertsias G, Ishigatsubo Y, Mizuki N, Tugal-Tutkun I, Seyahi E, Ozyazgan Y, Sacli FS, Erer B, Inoko H (2013). Genome-wide association analysis identifies new susceptibility loci for Behcet’s disease and epistasis between HLA-B*51 and ERAP1. Nat Genet.

[4] Badran YR, Dedeoglu F, Leyva Castillo JM, Bainter W, Ohsumi TK, Bousvaros A, Goldsmith JD, Geha RS, Chou J (2017). Human RELA haploinsufficiency results in autosomal-dominant chronic mucocutaneous ulceration. J Exp Med.

[5] Zhou Q, Wang H, Schwartz DM, Stoffels M, Park YH, Zhang Y, Yang D, Demirkaya E, Takeuchi M, Tsai WL (2016). Loss-of-function mutations in TNFAIP3 leading to A20 haploinsufficiency cause an early-onset autoinflammatory disease. Nat Genet.

[6] Aeschlimann Florence A, Batu Ezgi D, Canna Scott W, Go Ellen, Gül Ahmet, Hoffmann Patrycja, Leavis Helen L, Ozen Seza, Schwartz Daniella M, Stone Deborah L, van Royen-Kerkof Annet, Kastner Daniel L, Aksentijevich Ivona, Laxer Ronald M (2018). A20 haploinsufficiency (HA20): clinical phenotypes and disease course of patients with a newly recognised NF-kB-mediated autoinflammatory disease. Annals of the Rheumatic Diseases.

[7] Berteau F, Rouviere B, Delluc A, Nau A, Le Berre R, Sarrabay G, Touitou I, de Moreuil C (2018). Autosomic dominant familial Behcet disease and haploinsufficiency A20: a review of the literature. Autoimmun Rev.

[8] Franco-Jarava C, Wang H, Martin-Nalda A, Alvarez SD, Garcia-Prat M, Bodet D, Garcia-Patos V, Plaja A, Rudilla F, Rodriguez-Sureda V (2018). TNFAIP3 haploinsufficiency is the cause of autoinflammatory manifestations in a patient with a deletion of 13Mb on chromosome 6. Clin Immunol.

[9] Zheng Cuifang, Huang Ying, Ye Ziqing, Wang Yuhuan, Tang Zifei, Lu Junping, Wu Jie, Zhou Ying, Wang Lin, Huang Zhiheng, Yang Haowei, Xue Aijuan (2018). Infantile Onset Intractable Inflammatory Bowel Disease Due to Novel Heterozygous Mutations in TNFAIP3 (A20). Inflammatory Bowel Diseases.

[10] Takagi M, Ogata S, Ueno H, Yoshida K, Yeh T, Hoshino A, Piao J, Yamashita M, Nanya M, Okano T (2017). Haploinsufficiency of TNFAIP3 (A20) by germline mutation is involved in autoimmune lymphoproliferative syndrome. J Allergy Clin Immunol.

[11] Lawless D, Pathak S, Scambler TE, Ouboussad L, Anwar R, Savic S (2018). A case of adult-onset Still’s disease caused by a novel splicing mutation in TNFAIP3 successfully treated with tocilizumab. Front Immunol.

[12] Kadowaki Tomonori, Ohnishi Hidenori, Kawamoto Norio, Hori Tomohiro, Nishimura Kenichi, Kobayashi Chie, Shigemura Tomonari, Ogata Shohei, Inoue Yuzaburo, Kawai Tomoki, Hiejima Eitaro, Takagi Masatoshi, Imai Kohsuke, Nishikomori Ryuta, Ito Shuichi, Heike Toshio, Ohara Osamu, Morio Tomohiro, Fukao Toshiyuki, Kanegane Hirokazu (2018). Haploinsufficiency of A20 causes autoinflammatory and autoimmune disorders. Journal of Allergy and Clinical Immunology.

[13] Ohnishi H, Kawamoto N, Seishima M, Ohara O, Fukao T (2017). A Japanese family case with juvenile onset Behcet’s disease caused by TNFAIP3 mutation. Allergol Int.

[14] Shigemura T, Kaneko N, Kobayashi N, Kobayashi K, Takeuchi Y, Nakano N, Masumoto J, Agematsu K (2016). Novel heterozygous C243Y A20/TNFAIP3 gene mutation is responsible for chronic inflammation in autosomal-dominant Behcet’s disease. RMD Open.

[15] Duncan Christopher J A, Dinnigan Emma, Theobald Rachel, Grainger Angela, Skelton Andrew J, Hussain Rafiqul, Willet Joseph D P, Swan David J, Coxhead Jonathan, Thomas Matthew F, Thomas Julian, Zamvar Veena, Slatter Mary A, Cant Andrew J, Engelhardt Karin R, Hambleton Sophie (2017). Early-onset autoimmune disease due to a heterozygous loss-of-function mutation inTNFAIP3(A20). Annals of the Rheumatic Diseases.

[16] Mizushima YIG, Mimura Y, Ono S (1987). Guide for diagnosis of Behçet’s disease.

[17] Kirino Y, Ideguchi H, Takeno M, Suda A, Higashitani K, Kunishita Y, Takase-Minegishi K, Tamura M, Watanabe T, Asami Y (2016). Continuous evolution of clinical phenotype in 578 Japanese patients with Behcet’s disease: a retrospective observational study. Arthritis Res Ther.

[18] Iwama K, Iwata A, Shiina M, Mitsuhashi S, Miyatake S, Takata A, Miyake N, Ogata K, Ito S, Mizuguchi T (2018). A novel mutation in SLC1A3 causes episodic ataxia. J Hum Genet.

[19] Miyatake S, Koshimizu E, Fujita A, Fukai R, Imagawa E, Ohba C, Kuki I, Nukui M, Araki A, Makita Y (2015). Detecting copy-number variations in whole-exome sequencing data using the eXome Hidden Markov Model: an ‘exome-first’ approach. J Hum Genet.

[20] Tsuchida N, Nakashima M, Kato M, Heyman E, Inui T, Haginoya K, Watanabe S, Chiyonobu T, Morimoto M, Ohta M (2018). Detection of copy number variations in epilepsy using exome data. Clin Genet.

[21] Fromer M, Moran JL, Chambert K, Banks E, Bergen SE, Ruderfer DM, Handsaker RE, McCarroll SA, O'Donovan MC, Owen MJ (2012). Discovery and statistical genotyping of copy-number variation from whole-exome sequencing depth. Am J Hum Genet.

[22] Nord AS, Lee M, King MC, Walsh T (2011). Accurate and exact CNV identification from targeted high-throughput sequence data. BMC Genomics.

[23] Hug N, Longman D, Caceres JF (2016). Mechanism and regulation of the nonsense-mediated decay pathway. Nucleic Acids Res.

[24] Richards S, Aziz N, Bale S, Bick D, Das S, Gastier-Foster J, Grody WW, Hegde M, Lyon E, Spector E (2015). Standards and guidelines for the interpretation of sequence variants: a joint consensus recommendation of the American College of Medical Genetics and Genomics and the Association for Molecular Pathology. Genet Med.

[25] Ihrie RA, Marques MR, Nguyen BT, Horner JS, Papazoglu C, Bronson RT, Mills AA, Attardi LD (2005). Perp is a p63-regulated gene essential for epithelial integrity. Cell.

[26] Ihrie RA, Bronson RT, Attardi LD (2006). Adult mice lacking the p53/p63 target gene Perp are not predisposed to spontaneous tumorigenesis but display features of ectodermal dysplasia syndromes. Cell Death Differ.

[27] Koné-Paut I, Shahram F, Darce-Bello M, Cantarini L, Cimaz R, Gattorno M, Anton J, Hofer M, Chkirate B, Bouayed K (2016). Consensus classification criteria for paediatric Behçet’s disease from a prospective observational cohort: PEDBD. Ann Rheum Dis.

[28] Canna SW, de Jesus AA, Gouni S, Brooks SR, Marrero B, Liu Y, DiMattia MA, Zaal KJ, Sanchez GA, Kim H (2014). An activating NLRC4 inflammasome mutation causes autoinflammation with recurrent macrophage activation syndrome. Nat Genet.

[29] Romberg N, Al Moussawi K, Nelson-Williams C, Stiegler AL, Loring E, Choi M, Overton J, Meffre E, Khokha MK, Huttner AJ (2014). Mutation of NLRC4 causes a syndrome of enterocolitis and autoinflammation. Nat Genet.

