# Supplementary material for: Haploinsufficiency of A20 caused by a novel nonsense variant or entire deletion of TNFAIP3 is clinically distinct from Behçet’s disease
Source: Arthritis Res Ther. 2019 Jun 4;21:137. doi: 10.1186/s13075-019-1928-5 (PMC6549368; doi:10.1186/s13075-019-1928-5)
Supplement: Supplementary file 1 — Table S1. Clinical features of Japanese haploinsufficiency of A20 (HA20) and Japanese Behçet’s disease (BD). (DOCX 22 kb) [file 13075_2019_1928_MOESM1_ESM.docx]

**Table S1. Clinical features of Japanese haploinsufficiency of A20 (HA20) and Japanese Behçet’s disease (BD)**

| Characteristics | HA20 ^10,12-14^  (n=26 ^a^) | (%) | BD  (n=514) | (%) | p | Odds ratio | 95%CI | |
| --- | --- | --- | --- | --- | --- | --- | --- | --- |
| Age at onset ^b^ (years) (mean ± SD) | 5.08 ± 5.48 |  | 36.38 ± 12.28 |  | **< 0.001** ^f^ |  |  |  |
| Childhood onset (< 16 years old) | 25 /26 | 96.2 | 10 /514 | 1.9 | **< 0.001** | 1260.00 | 155.15 | 10232.83 |
| Observation period (years) (mean ± SD) | 16.61 ± 16.51 |  | 13.65 ± 12.01 |  | 0.28 ^f^ |  |  |  |
| Gender male | 10 /26 | 38.5 | 239 /514 | 46.5 | 0.42 | 0.72 | 0.32 | 1.62 |
| Familial ^c^ | 8 /11 | 72.7 | 19 /327 | 5.8 | **< 0.001** | 43.23 | 10.60 | 176.26 |
| Recurrent fever | 20 /26 | 76.9 | 39 /359 | 10.9 | **< 0.001** | 27.35 | 10.36 | 72.22 |
| Oral ulcer | 21 /26 | 80.8 | 512 /514 | 99.6 | **< 0.001** | 0.02 | 0.00 | 0.09 |
| Genital ulcer | 15 /26 | 57.7 | 366 /514 | 71.2 | 0.14 | 0.55 | 0.25 | 1.23 |
| Eye involvement | 1 /26 | 3.9 | 328 /514 | 63.8 | **< 0.001** | 0.02 | 0.00 | 0.17 |
| Skin involvement | 11 /26 | 42.3 | 456 /514 | 88.7 | **< 0.001** | 0.09 | 0.04 | 0.21 |
| Arthritis | 4 /26 | 15.4 | 242 /514 | 47.1 | **0.002** | 0.20 | 0.07 | 0.69 |
| Gastrointestinal involvement ^d^ | 12 /26 | 46.1 | 75 /514 | 14.6 | **< 0.001** | 5.02 | 2.23 | 11.27 |
| Vascular involvement | 2 /26 | 7.7 | 41 /514 | 8.0 | 1.00 | 0.96 | 0.22 | 4.21 |
| CNS involvement | 1 /26 | 3.9 | 57 /514 | 11.1 | 0.34 | 0.32 | 0.04 | 2.41 |
| Autoimmune diseases ^e^ | 12 /26 | 46.1 | 36 /390 | 9.2 | **< 0.001** | 8.43 | 3.63 | 19.60 |
| Anti-nuclear antibody (≧160x) | 0 /1 | 0.0 | 15 /284 | 5.3 | 1.00 | - | - | - |
| HLA-B51 | 1 /6 | 16.7 | 193 /404 | 47.8 | 0.22 | 0.22 | 0.03 | 1.89 |
| Past/current colchicine use | 15 /26 | 57.7 | 368 /512 | 71.9 | 0.12 | 0.53 | 0.24 | 1.19 |
| Past/current bDMARDs use | 6 /26 | 23.1 | 88 /514 | 17.1 | 0.44 | 1.45 | 0.57 | 3.72 |
| Fulfilling ISG criteria for BD | 10 /26 | 38.5 | 462 /514 | 89.9 | **< 0.001** | 0.07 | 0.30 | 0.16 |

BD, Behçet’s disease; bDMARDs, biological disease modifying anti-rheumatic drugs; CNS, central nervous system; HA20, haploinsufficiency of A20; ISG, International Study Group. Significant results are highlighted in bold.

^a^ Includes previously reported ^10,12-14^ and current cases with genetically confirmed HA20.

^b^ Age at ‘symptom onset’ for HA20 and ‘diagnosis’ for BD.

^c^ Ratio of pedigree with familial aggregation among all families.

^d^ Cases with gastrointestinal lesions revealed by imaging (endoscope, CT).

^e^ Autoimmune diseases including systemic diseases (rheumatic diseases) and organ specific diseases (ex. Hashimoto disease, Insulin dependent diabetes etc.)

^f^ Analyzed using the unpaired t test. Other variables were analyzed using the chi-square test.
