# Supplementary material for: Haploinsufficiency of A20 caused by a novel nonsense variant or entire deletion of TNFAIP3 is clinically distinct from Behçet’s disease
Source: Arthritis Res Ther. 2019 Jun 4;21:137. doi: 10.1186/s13075-019-1928-5 (PMC6549368; doi:10.1186/s13075-019-1928-5)
Supplement: Supplementary file 2 — Table S2. Clinical features of haploinsufficiency of A20 (HA20) and the Pediatric Behçet’s disease (PEDBD). (DOCX 21 kb) [file 13075_2019_1928_MOESM2_ESM.docx]

**Table S2. Clinical features of haploinsufficiency of A20 (HA20) and the Pediatric Behçet’s disease (PEDBD)**

| Characteristics | HA20 ^5-15^  (n=54 ^a^) | (%) | PEDBD ^27^  (n=156 ^b^) | (%) | p | Odds ratio | 95%CI | |
| --- | --- | --- | --- | --- | --- | --- | --- | --- |
| Age at symptom onset (years) (mean ± SD) | 6.0 ± 6.5 |  | 7.8 ± 4.4 |  | - |  |  |  |
| Childhood onset (< 16 years old) | 49 /53 | 92.5 | 156 /156 | 100 | **0.004** | - | - | - |
| Observation period (years) (mean ± SD) | 15.5 ± 14.9 |  | 7.1 ± 3.6 |  | - |  |  |  |
| Gender male | 20 /54 | 37.0 | 78 /156 | 50.0 | 0.10 | 0.59 | 0.31 | 1.11 |
| Familial ^c^ | 15 /25 | 60.0 | 32 /156 | 20.5 | **< 0.001** | 5.81 | 2.39 | 14.15 |
| Recurrent fever | 37 /51 | 72.5 | 68 /156 | 43.6 | **< 0.001** | 3.42 | 1.71 | 6.83 |
| Oral ulcer | 46 /52 | 88.5 | 156 /156 | 100 | **< 0.001** | - | - | - |
| Genital ulcer | 34 /52 | 65.4 | 86 /156 | 55.1 | 0.20 | 1.54 | 0.80 | 2.95 |
| Eye involvement | 5 /52 | 9.6 | 71 /156 | 45.5 | **< 0.001** | 0.13 | 0.05 | 0.34 |
| Skin involvement | 28 /52 | 53.8 | 104 /156 | 66.7 | 0.10 | 0.58 | 0.31 | 1.11 |
| Arthritis | 21 /54 | 38.9 | 64 /156 | 41.0 | 0.78 | 0.92 | 0.49 | 1.72 |
| Gastrointestinal involvement ^d^ | 34 /53 | 64.2 | 46 /156 | 29.5 | **< 0.001** | 4.28 | 2.22 | 8.27 |
| Vascular involvement | 7 /53 | 13.2 | 23 /156 | 14.7 | 0.78 | 0.88 | 0.35 | 2.19 |
| CNS involvement ^d^ | 5 /53 | 9.4 | 93 /156 | 59.6 | **< 0.001** | 0.03 | 0.03 | 0.19 |
| Fulfilling ISG criteria for BD | 23 /54 | 42.6 | 115 /156 | 73.7 | **< 0.001** | 0.27 | 0.14 | 0.51 |
| Fulfilling PEDBD criteria for BD | 26 /54 | 48.1 | 143 /156 | 91.7 | **< 0.001** | 0.08 | 0.04 | 0.18 |

BD, Behçet’s disease; CNS, central nervous system; HA20, haploinsufficiency of A20; ISG, International Study Group; PEDBD, Pediatric Behçet’s disease. Significant results are highlighted in bold. Analysed using the chi-square test.

^a^ Includes previously reported ^5-15^ and current cases with genetically confirmed HA20.

^b^ Includes Confirmed BD cases in PEDBD ^27^.

^c^ Ratio of pedigree with familial aggregation among all families.

^d^ Cases with gastrointestinal signs include cases with abdominal pain and CNS signs include those with headaches
